# Association between dietary antioxidant index and risk of Helicobacter pylori infection among adults: a case–control study

**DOI:** 10.1186/s12876-022-02488-3

**Published:** 2022-09-06

**Authors:** Zohreh Ebrahimi, Mohsen Masoodi, Zahra Aslani, Sina Naghshi, Masoumeh Khalighi Sikaroudi, Farzad Shidfar

**Affiliations:** 1 Department of Nutrition, School of Public Health, Iran University of Medical Sciences, Tehran, Iran; 2 Colorectal Research Center, Rasoul-e-Akram Hospital, Iran University of Medical Sciences, Tehran, Iran; 3 The Ohio State University Interdisciplinary ph.D. program in Nutrition (OSUN), Columbus, USA; 4 Students’ Scientific Research Center, Tehran University of Medical Sciences, Tehran, Iran; 5 Department of Clinical Nutrition, School of Nutritional Sciences and Dietetics, Tehran University of Medical Sciences, Tehran, Iran; 6 The Ohio State University Comprehensive Cancer Center, Columbus, USA

**Keywords:** Helicobacter pylori, Dietary antioxidant index, Antioxidants

## Abstract

**Backgrounds and aims:**

One of the most important risk factors for Helicobacter pylori (H. pylori) infection is nutrition. Balanced diets with high antioxidant properties may have protective effects against the consequences of this infection. In the current study, we aimed to investigate the association between the dietary antioxidant index and the risk of H. pylori infection among adults.

**Methods:**

In a case–control study the dietary intake of patients with H. pylori infection was compared with healthy subjects. The dietary antioxidant index (DAI) was calculated using dietary intakes derived from a validated food frequency questionnaire (FFQ). Demographic information was obtained by a related questionnaire and Physical Activity was measured by International Physical Activity Questionnaire (IPAQ) were used to obtain information. Using logistic regression models, we evaluated the association between the DAI and H. pylori infection risk. The significance level was determined as *P* < 0.05.

**Results:**

Finally, dietary data of 148 cases and 302 controls (mean age: 38.72 ± 10.61 (were analyzed. The mean of total DAI was significantly higher in controls (7.67) when compared with H. pylori cases (3.57) (*P* < 0.001). After adjustment for covariates, participants with less than median DAI values had an increased risk of H. pylori onset (adjusted OR 1.08, 95% CI: 1.02–1.12, *P* < 0.001).

**Conclusions:**

Appropriate intake of nutrient antioxidants may have a role in decreasing the likelihood of H. pylori infection risk**.**

## Introduction

*Helicobacter pyl*ori (*H. pylori*) is a gram-negative, microaerophilic and spiral shape pathogen, which replicates in the gastric epithelium layer and can be transferred directly (oral–oral) or indirectly (fecal–oral) to another. [1]. Approximately, there were 4.4 billion individuals with *H. pylori* infection worldwide [2]. In a study conducted in Jordan, Iran, Russia, China, Canada and Latin American countries, a large outbreak of *H. pylori* infection was reported [3]. This bacterium recognized to main factor of chronic gastritis, peptic ulcer and primary gastric lymphoma and according to the definition of the International Association for Cancer Research (IARC) type I carcinogenic agent [4]. The pathogenic effect of Helicobacter pylori infection is more in the upper gastrointestinal tract. Helicobacter pylori can indirectly affect the brain-gut axis in addition to its direct proinflammatory and cytotoxic effects [5].

Along other risk factors, nutrition and nutritional status play an important role in Helicobacter pylori infection. Therefore, a balanced diet, especially high consumption of fruits and vegetables, might protects people versus he consequences of *H. pylori* infection [6]. In a study that assessed the association of dietary patterns with *H. pylori* infection, high intake of grain and vegetables, was inversely related to Helicobacter pylori infection [7]. On the contrary, a diet full of carbohydrates and sweets was related to a higher *H. pylori* infection prevalence [8].

Enough intake of dietary antioxidants predominantly from fruits and vegetables can provide a balanced defense mechanism against the harmful effects of reactive oxygen species [9]. Bacterial growth is inhibited by antioxidants through reducing reactive oxygen species and inflammations caused by *H. pylori* [10]. Studies have indicated that *H. pylori* infection is related to the deficiencies in vitamin A, C, and E [11–14]. Moreover, vitamins C and E has shown an inhibitory effect on *H. pylori* intensity and neutrophilic activity [15, 16]. The gastric epithelium damage which is caused by *H. pylori* has a direct relationship with reduced concentration of vitamin C [9]. Several studies have shown that concentration of vitamins C and E are lower in peoples with *H. pylori* than in those without *H. pylori* [17–21].

The dietary antioxidant index (DAI) has become a valid indicator that considers total dietary antioxidant properties at once. As the importance of dietary antioxidants has been shown in the prevention and control of *H. pylori* infection, the total dietary antioxidant capacity can be evaluated by the DAI in these patients. Based on our knowledge the present study is the first study which was planned and conducted to investigate the relation to between DAI and *H. pylori* infection.

## Materials and methods

### Study design and participants

A total of 148 patients with *H. pylori* infection and 302 *H. pylori* negative subjects participated in the case–control study. The participants in the case group (*H. pylori* positive) were recruited from the Gastroenterology clinic, Rasoul-e-Akram Hospital, Tehran, Iran; between June 2021 and November 2021. These patients were newly diagnosed (active phase of the infection) with *H. pylori* and were not under treatment prior to participation in this study. For *H. pylori* infection diagnosis, we used one of the following methods: blood antibody, fecal antigen, urea breath test (UBT), endoscopy and gastric biopsy. The control group was twice as numerous as the case group and comprised patients referred to other sections of this hospital (ophthalmology, orthopedics, maxillofacial surgery, and otorhinolaryngology) which had no infection with *H. pylori* based on only one of the following diagnostic methods: blood antibody, fecal antigen, urea breath test (UBT), endoscopy and gastric biopsy. All the H. pylori negative subjects who entered the study were new cases. In addition, people who had Helicobacter before and were treated or who are resistant to treatment were not included in the control group and were excluded from the study. Participants who had a medical history of certain diseases (gastrointestinal cancers, psychosis or diseases that would result in memory disturbances), pregnancy, lactating and having an arbitrary special diet considered as an exclusion criteria. Also, individuals who over-or under-estimated their energy intake (> 4200 or < 800 kcal/d) were excluded [22]. Cases and controls were matched for age, sex, and smoking.

### Anthropometric assessment

Anthropometric measurements were conducted by a trained dietician. Weight was measured with minimum clothing, without shoes, by using a digital scale (Seca 807, made in Germany) (100 g accuracy). Standing height measurement was done without shoes, flat feet against a wall, arms at sides, looking straight by a stadiometer (Seca 206) (0.5 cm accuracy). Body mass index (BMI) was calculated as weight (kg) divided by height in square meters (m^2^).

### Assessment of other variables

Data on age, sex (male/female), smoking (yes/no), and alcohol consumption (yes/no) of the participants were collected through face-to-face interviews by related questionnaires. We used the International Physical Activity Questionnaire (IPAQ) to measure the current physical activity of participants through face-to-face interviews [23].

### Dietary assessment and dietary antioxidant index (DAI)

Dietary intakes were assessed using a validated block-format 168-item food frequency questionnaire (FFQ) in the preceding year [24]. Two experienced interviewers fulfilled FFQ. Based on this questionnaire, participants were asked to report their dietary intakes in a format of day, week, month, or year. Moreover, they should report their intakes based on the serving size of each food item. To increase the accuracy of estimates, interviewers showed household measurements or serving size of each food item to participants. Finally, based on each item’s consumption frequency and serving size, we calculated the gr/day intake of each food item. In addition, we calculated the daily nutrients intake for each participant according to the nutrient contents of foods.

We also calculated DAI for the participants based on FFQ data. We utilized previously published databases from the USA and Italy, containing the most commonly consumed foods for calculation of DAI [25]. The validity of this index was measured before [26]. We used the method which recommended by Benzie for measuring the total antioxidant capacity of the participants’ food intake (their dietary antioxidant index) [27]. To obtain the DAI, we standardized each of vitamin A, C, E, selenium, manganese, and zinc by subtracting the value of global mean intake of each dietary antioxidant factor from reported consumed amount. Then, this number was divided by world standard deviation. Finally, the DAI was calculated by summing the standardized intakes, as described below.

$${\text{DAI}} = \mathop \sum \limits_{{{\text{i}} = 1}}^{n = 6} \frac{{{\text{Individual}}\;\;{\text{ intake }} - {\text{ Mean}}}}{SD}$$

### Statistical analysis

Statistical analysis was conducted using Statistical Package Software for Social Science, version 21 (SPSS Inc., Chicago, IL, USA). The Kolmogorov–Smirnov’s test and histogram chart were used to test the normality of the data. Baseline characteristics and dietary intakes were expressed as mean ± SD or median (interquartile range (IQR)) for quantitative variables with normal and skewed distribution, respectively. Furthermore, the number (percentage) was used to indicate the qualitative variables. Comparison of the data between two groups was done using independent sample t-test and chi square for continuous and categorical variables, respectively. Binary logistic regression was used to estimate odds ratios (ORs) and 95% confidence intervals (CIs) adjusted for multiple covariates in different models. The significance level was determined as *P* < 0.05.

## Result

Among 153 patients with *H. pylori* infection and 306 healthy subjects, finally, 148 case and 302 controls had completed information regarding dietary intakes and were included in the statistical analysis after excluding individuals who over-or under-estimated their energy intake. The mean age of study participants was 38.72 ± 10.61 years, and 59.8% were female. General characteristics of case and control groups are indicated in Table 1. Compared with those in the control groups, individuals with *H. pylori* infection were significantly older, had higher BMI and smoked more. No other significant difference was observed in. Other characteristics (Table 1).

**Table 1:** Baseline characteristics and hematological parameters of the study participant

Variables	Cases (148)	Controls (302)	*P* value
Age (years)	41 (32, 52.75)	36 (30, 43)	< 0.001
BMI	27.01 (23.87, 31.22)	24.67(22.53, 26.87)	< 0.001
Male, N(%)	46 (31.1)	135 (44.7)	0.004
Current smokers, N (%)	11 (7.4)	8 (2.6)	0.019
Alcohol consumption, N (%)	16 (10.8)	21 (7.0)	0.113
*Physical activity, N (%)*			
Low	113 (76.3)	220 (72.8)	< 0.001
Moderate	29 (19.6)	59 (19.5)	
High	6 (4.1)	23 (7.6)	

Data are presented as mean ± SD or percent (N)

*BMI* body mass index

Obtained from the analysis of independent-samples T Test or chi-squared test, where appropriate

As we show in Table 2, individuals with *H. pylori* had a lower intake of vitamin E, vitamin A, manganese, and selenium compared to controls. Other differences in dietary intakes among the cases and controls were not significant.

**Table 2:** Dietary intake among the case and control groups

Variables	Cases (148)	Controls (302)	*P* value
Vitamin E (mg/d)	10.48 (7.99,13.96)	12.46 (9.65, 15.45)	0.001
Vitamin A (RAE/d)	402.85(283.95, 568.83)	553 (370.29, 786)	< 0.001
Vitamin C (mg/d)	132 (86.76, 169.38)	112 (75.76, 159.38)	0.334
Zinc (mg/d)	13.53 (10.88, 16.87)	10.49 (8.69, 12.83)	0.124
Manganese (mg/d)	6.32 (4.91,8.02)	10.20 (7.49,13.28)	< 0.001
Selenium (mg/d)	103.44 (81.75, 129.88)	130.85 (98.97, 174.36)	< 0.001

Data are presented as median (interquartile range )IQR()

Obtained from the analysis of 2-independent- samples Tests

The mean of total DAI was significantly higher in controls (7.67) when compared with *H. pylori* cases (3.57) (*P* < 0.001). The crude and adjusted ORs (95% CIs) for the underlying associations between both continuous and categorical DAI with *H. pylori* risk have been shown in Table 3. We observed a significant decrease in the *H. pylori* risk (OR 0.82, 95% CI: 0.75–0.89, *P* < 0.001) when continuous DAI was increased. Participants with less than median DAI values had increased risk of *H. pylori* onset (adjusted OR 1.08, 95% CI: 1.02–1.12, *P* < 0.001).

**Table 3:** Association between DAI and H. pylori risk

	Case (148)	Control (302)	Unadjusted OR (95% CI)	P-value*	Adjusted^a^ OR (95% CI)	*P* value
Total DAI, mean (SD)	3.57 (1.55)	7.67 (2.74)	0.87 (0.81, 0.92)	< 0.001	0.82 (0.75, 0.89)	< 0.001
> Median, N(%)	102 (22.7)	123 (27.3)	1	–	1	–
≤ Median, N(%)	46 (10.2)	179 (39.8)	1.07 (1.04, 1.10)	< 0.001	1.08 (1.02, 1.12)	< 0.001

Data are presented as OR (95% CI)

*DAI* Dietary antioxidant index

*P for trend: obtained from binary logistic regression

^a^Adjusted for age, sex, smoking, alcohol, physical activity, BMI, education and total energy

## Discussion

In the current study, DAI as a continuous variable was inversely associated with *H. pylori* infection. We also found a significant positive association between lower DAI and odds of *H. pylori* infection in functional categories (≤ Median vs. > Median). *H. pylori* is one of the most common bacterial infections in human beings. The role of *H. pylori* infection in gastric cancers and other gastrointestinal tract diseases has been widely established [28]. Available evidence indicates that diet has important role in developing *H. pylori* infection. Therefore, protective dietary factors are important from a public health point of view. While some nutritional research has widely focused on single nutrients or foods in diet-disease relations, the overall diet could be more informative because humans typically consume a combination of nutrients and foods. Dietary indices such as DAI are one of the approaches for this purpose which considers the whole quality of the diet. A significant inverse association has been reported between DAI and some diseases with inflammatory nature, including gastric cancer [29], colorectal cancer [30], nonalcoholic fatty liver [31], and obesity [32]. To our knowledge, no previous studies have investigated DAI in relation to *H. pylori* infection. The results from this study are consistent with a cross-sectional study, which found healthy subjects significantly have higher intakes of fruits, vegetables, and vitamin C compared to subjects with *H. pylori* infection. However, no significant differences were observed in vitamin A and E and zinc intake [33]. In another study that examined the association of dietary factors with H pylori re-infection, high consumption of fruit and vegetables, which contains vitamin C, was associated with decreased risk of H pylori re-infection [34]. In a cross-sectional study on 1106 men and women, plasma vitamin C level for *H. pylori*-infected patients was only 80% of that for non-infected individuals [18]. Similar findings were also seen in the study of Annibal et al., in which plasma ascorbic acid concentrations were lower in *H. pylori* -infected groups than in healthy controls [35]. Finding from a small randomized clinical trial showed that Zinc L-carnosine supplements in combination with three medications (lansoprazole, amoxicillin, clarithromycin) could significantly improve the cure rate for *H. pylori* [36]. It has been shown that *H. pylori*-infected patients with non-atrophic chronic gastritis had lower zinc concentrations in gastric mucosa than uninfected patients with the same type of gastritis [37]. Our findings were also consistent in line with evidence from animal studies. For example, one study reported that high doses of vitamin C could inhibit the in vitro and in vivo colonization of *H. pylori* [38]. In an animal study, a high intake of selenium, β-Carotene, and vitamins A, C, and E in the Guinea Pig led to a significant reduction in *H. pylori* growth [39]. Two further animal studies reported that zinc supplementation inhibits *H. pylori* -induced gastric mucosal oxidative inflammation in gerbils [40, 41]. In contrast to our findings, two meta-analyses of randomized controlled trials reported that supplementation with vitamins C and/or E could not improve the eradication rate of *H. pylori* [42, 43]. The reason that high doses of vitamin C is not able to eradicate *H. pylori* infection might be explained by the fact that at chronic infection, some patients develop relative achlorhydria that leads to lower acidity of gastric juice [44]. In such conditions, the anti-urease role of vitamin C in eradicating *H. pylori* is relatively less important. Furthermore, the complex mixture of diet-deriving nutrients may be more effective than high doses of single micronutrient supplements. It is possible that such nutrients interact or work together but have smaller effects individually [45]. Moreover, this non-significant effect may be partly due to the small sample size, limited duration of intervention, and low to moderate methodological quality of some of the *H. pylori* RCTs. Therefore caution is needed in interpreting the results of such studies.

Several biologically plausible reasons may explain why dietary antioxidants might be, either directly or indirectly, a protective factor againstH.pylori infection. It is well-known that antioxidants, with their free radical scavenging activities, can inhibit the growth of *H. pylori* [39]. *H. pylori* is urease positive and can synthesize a large amount of urease for ammonia production to neutralize the gastric acid, allowing it to colonize in the stomach epithelium [46]. It has been shown that vitamin C inhibits urease activity [47]. Moreover, vitamin C improves the stimulation and activity of granulocytes, macrophages, lymphocytes, and immunoglobulin production [34, 48]. In vitro studies, showed that zinc inhibits the urease enzyme and prevents H.pylori adhesion to gastric mucin. Mucosal inflammation may be required for *H. pylori* infection to persist, and the anti-inflammatory effects of antioxidants could inhibit *H. pylori* growth [39, 49].

## Strengths and limitations

This study included both strengths and weaknesses that should be noted. Our study is the first report to investigate the association of DAI and *H. pylori* infection; it has high scientific competence and novelty level. Moreover, results were significant in different statistical approaches (continuous and categorical). We also used valid and reliable questionnaires for data collection, which can further support the accuracy of findings. The nearly complete participation of identified cases and controls is another strength of our study. Limitations of the current study are generic concerns for case–control studies. The causality of the associations cannot be established from this study. Because we used FFQ for dietary assessment, inaccurate measures of dietary intakes may have resulted in misclassification. As participants reported past dietary habits, recall bias may have occurred, particularly among patients, as changes in lifestyle may occur when *H. pylori* infection develops and becomes symptomatic. Nevertheless, we included newly diagnosed patients who were interviewed by uniformly trained interviewers in their hospital settings and were unaware of any study hypothesis to minimize changes in dietary habits. It is possible that selection bias may have resulted from using hospital-based controls since these controls include individuals with conditions that could potentially be related to their dietary antioxidants intake. Moreover, the significant association between DAI and *H. pylori* infection could be due to unmeasured or residual confounding. Finally, we did not have data on cooking methods of antioxidant-rich foods that could alter the antioxidant content of such foods.

## Conclusion

This case–control study provides evidence for an inverse association between higher DAI and odds of *H. pylori* infection. More in-depth studies are needed to confirm our findings.

## Data Availability

The datasets used or analyses during the current study available from the corresponding author on reasonable request.

## Abbreviations

BMI: Body mass intake
CI: Confidence interval
DAI: Dietary antioxidant index
FFQ: Food frequency questionnaire
*H. pylori*: Helicobacter pylori
IPAQ: International Physical Activity Questionnaire
OR: Odds ratio
IARC: International Association for Cancer Research
IQR: Interquartile range
SD: Standard deviation
